# Reduced vestibular perception thresholds in persistent postural-perceptual dizziness- a cross-sectional study

**DOI:** 10.1186/s12883-021-02417-z

**Published:** 2021-10-12

**Authors:** Sebastian Wurthmann, Dagny Holle, Mark Obermann, Miriam Roesner, Michael Nsaka, Armin Scheffler, Christoph Kleinschnitz, Steffen Naegel

**Affiliations:** 1 Department of Neurology and Dizziness and Vertigo Center Essen, University of Duisburg-Essen, Hufelandstr. 55, 45147 Essen, Germany; 2 Center for Translational Neuro- and Behavioral Sciences, University of Duisburg-Essen, Essen, Germany; 3 Department of Neurology, Weser-Egge Hospital Höxter, University of Duisburg-Essen, Höxter, Germany; 4 Department of Neurology, Martin-Luther-University Halle-Wittenberg, Halle/Saale, Germany

**Keywords:** Persistent postural-perceptual dizziness, functional vestibular disorder, vestibular perceptual threshold, motion sensitivity

## Abstract

**Background:**

Persistent postural-perceptual dizziness (PPPD) is the most common functional vestibular disorder. A multisensory mismatch altered by psychological influences is considered to be an important pathophysiological mechanism. Increased cortical and subcortical excitability may play a role in the pathophysiology of PPPD. We hypothesized that decreased motion perception thresholds reflect one mechanism of the abnormal vestibular responsiveness in this disorder. We investigated the vestibular perception thresholds and the vestibular ocular reflex with a rotatory chair experiment to gain insights in the processing and adaption to vestibular provocation.

**Methods:**

In this cross-sectional study 26 female PPPD patients and 33 healthy female age matched controls (HC) were investigated sitting in a motorized rotary chair shielded regarding visual and acoustic stimuli. The chair was rotated for 20 minutes with slowly increasing velocity to a maximum of 72°/s. We functionally tested motion perception thresholds and vegetative responses to rotation as well as vestibular-ocular reflex thresholds. We additionally investigated several psychological comorbidities (i.e. depression, anxiety, somatosensory amplification) using validated scores. Conventional dizziness scores were obtained to quantify the experienced dizziness and impact on daily life.

**Results:**

PPPD patients showed a significant reduced vestibulo-perceptual threshold (PPPD: 10.9°/s vs. HC: 29.5°/s; *p*<0.001) with increased motion sensitivity and concomitant vegetative response during and after the chair rotation compared to healthy controls. The extent of increased vestibular sensitivity was in correlation with the duration of the disease (*p*=0.043). No significant difference was measured regarding nystagmus parameters between both groups.

**Conclusion:**

PPPD patients showed increased vegetative response as well as decreased vestibulo-perceptual thresholds which are related to disease duration. This is of interest as PPPD might be sustained by increased vestibular excitability leading to motion intolerance and induction of dizziness when exposed to movement.

**Supplementary Information:**

The online version contains supplementary material available at 10.1186/s12883-021-02417-z.

## Introduction

Persistent postural-perceptual dizziness (PPPD) is a common chronic functional vestibular disorder predominantly in middle-aged patients [1]. As early as 1975, first reports described several syndromes of spatial disorientation and altered sensations of motion, including supermarket-syndrome [2] and visually induced motion [3]. It was then termed phobic postural vertigo (PPV) by Brandt and Dieterich [4]. Staab et al. further refined the concept of this syndrome and renamed it to chronic subjective dizziness (CSD) [5]. In 2017, a consensus document of the Bárány-Society (The International Society for Neuro-Otology) with new diagnostic criteria was published terming the disorder “persistent postural-perceptual dizziness”, which will be added to the International Classification of Diseases (ICD-11) [6].

PPPD affects men and women at all ages. Several clinical epidemiological studies have been published worldwide and showed a female predominance in PPPD [7–9]. PPPD is characterized by persisting subjective dizziness, unsteadiness or non-rotational vertigo for at least three months and may be exacerbated by upright posture including the patient’s own movement as well as motion of complex visual stimuli [10].

It often follows somatic vestibular disorders such as benign paroxysmal positional vertigo (BPPV), vestibular migraine, or Menière’s disease, as well as other medical or psychiatric events associated with balance-related problems [6].

The exact pathophysiologic mechanisms of PPPD remain unclear. Patients suffering PPPD do not only have trouble with balance and motion but also have poorer navigational abilities [11]. This may also lead to difficulties in psychological coping mechanisms or inefficient adaptation techniques.

Failure of adaptation of the postural control system following neuro-otologic diseases or other dizziness-related conditions was suggested as a pathophysiological hallmark for the development of PPPD. Eventually, one central feature is the heightened sensitivity to motion [6].

Thus, e we hypothesize a lowered motion perception threshold, reflecting one aspect of the abnormal vestibular responsiveness in this disorder.

The purpose of our study was to investigate the vestibular perceptual threshold for a vestibular specific stimulus (passive body rotation in yaw plane), physiological vestibular reflexes (e.g. the vestibulo-ocular threshold), and the development of motion sickness in patients with PPPD and controls.

## Methods

### Participants

Twenty-six females suffering from PPPD were recruited in the Dizziness and Vertigo Center in Essen. All patients fulfilled the diagnostic criteria of PPPD according to the Bárány-Society [6]. Since the willingness of females to participate in this experiment was much higher than that of males, we decided to unify the cohort by only including females. PPPD patients were compared to 33 age- and gender-matched healthy subjects in a cross-sectional study design. The healthy controls were recruited from the local community after excluding those with a history of dizziness or vertigo, migraine or other neurological and psychiatric disorders. No participant was investigated by the rotatory chair ever before.

This study was conducted in accordance with the Declaration of Helsinki and its later amendments. The study protocol was approved by the local Ethics Committee of the University of Duisburg-Essen. Informed consent for participation was obtained from all participants.

### Assessment and examination

All participants were interviewed face-to-face regarding their symptoms and demographic data were obtained by a standardized questionnaire. All patients were systematically examined neurologically and neuro-otologically including examination with Frenzel goggles, positioning maneuvers, the Halmagyi-Curthoys head impulse test and the head-shaking test. Cervical vestibular evoked myogenic potentials (cVEMP) and videonystagmography analysis (VNG) including caloric testing were also assessed. Bithermal caloric responses were performed as part of the clinical workup in order to make a diagnosis. Participants had no identifiable structural deficits.

PPPD patients previously suffering benign paroxysmal positional vertigo were symptom free in this regard. Only patients with a fully recovered peripheral vestibular dysfunction and normal VNG were included. Exclusion criteria were any other active neuro-otologic and primary headache disorders. Psychiatric comorbidities such as anxiety and depression are common in PPPD [5]. PPPD patients with severe psychiatric comorbidities were not enrolled. Patients with relevant chronic somatic illness (e.g. diabetes, previous cardiovascular events, malignancies) were also excluded.

### Self-report measures

All participants answered the following structured questionnaires. Motion sickness in the past was evaluated by the Motion Sickness Susceptibility Questionnaire (MSSQ) [12]. Possible symptoms of depression or anxiety were assessed with the Hospital Anxiety and Depression Scale (HADS) [13] and the State-Trait Anxiety Inventory (STAI) [14]. Somatosensory amplification and health anxiety were investigated in this cohort by applying the Somatosensory Amplification Scale (SSAS) [15] and the Whitely Index (WI) [16]. In patients we conducted several questionnaires to quantify the intensity of the experienced dizziness and vertigo, as well as the impact of dizziness on daily life using the Vertigo Symptoms Scale (VSS) [17] and the Dizziness Handicap Inventory (DHI) [18].

### Rotary chair experiment

All participants were investigated seating in a motorized rotary chair (Nydiag 200, Interacoustics, Denmark), which was started with 0.1°/s and accelerated with 0.1°/s^2^. In total it took 12 minutes to achieve the maximum velocity of 72°/s on a vertical axis. The chair was stopped after max. 20 minutes. Alternatively the chair was stopped, when the participants developed severe dizziness or vegetative symptoms (sickness rating score 4, see below).

Habituation was previously reported to be altered in PPPD [19]. To avoid influences due to repetitive measurements, all participants were investigated only once rotated rightwards in horizontal (yaw) plane. The torso, legs and head were restrained to reduce proprioceptive information and all participants wore a video-oculography headset with a non-see-through system to avoid visual stimulation. In order to prevent effects of noise exposure participants wore noise-protection headphones.

Participants were instructed to press a button carrying in their hands when perceiving the beginning of motion. In the following participants were asked to verbally indicate the perceived rotation direction which was documented by the investigator. The time between the start of chair rotation and the button press was converted to degrees per second (°/s) and defined as the vestibular perception threshold.

Adapting a rotary chair paradigm of Murdin et al. participants were asked every minute during the rotation and during the recovery period at minute 1, 5, 10, 15 and 20 after the rotation ended to quantify nausea on a 4-point numeric sickness rating (SR) scale (1=no symptoms, 2=initial symptoms, but no nausea, 3=mild nausea, 4=severe dizziness, nausea or vomiting) [20]. We defined the “SR phase” as the duration of time participants rated their motion sickness according to a certain value on the SR scale. The recovery time began after the end of the 20-minute rotation or after entering SR phase 4.

The vestibular-ocular threshold and the per-rotatory nystagmus were recorded with Interacoustics VNG analysis software. Presence and beginning of the per-rotatory nystagmus were defined as a continuous and eye movement evaluated independently by three investigators (SW, SN and MR). Maximum and mean velocity of the slow phase (degrees/second) was calculated by a software (VN415, Interacoustics, Denmark). The same protocol was previously used and able to detect conclusive results in vestibular migraine [21].

### Statistical analyses

Statistical analysis was performed using IBM SPSS Statistics Version 23 (International Business Machines Corporation, Armonk, New York, USA). Parametric methods were applied for normally distributed metric variables and the non-parametric equivalents for all other variables. The analyses of the mean comparisons were performed using Student’s t-test or, for nonparametric data, the Mann-Whitney test. For ordinal data, such as the assignment to the individual phases after recovery time, Fishers-Exact-Test was used. Correlation analyses were carried out using Spearman rank correlation, as these data were not normally distributed. For all analyses a significance level of *p*<0.05 was applied.

## Results

### Demographic characteristics

The mean age did not differ between patients and controls (47.3 ±10.2 vs. 46.9 ±10.1 years, *p* = 0.902, t-test). The average duration of illness was 5.4 years ±5.5 years. Five PPPD patients had a history of a previous BPPV more than one year ago and one PPPD patient had a completely resolved vestibular neuritis also more than one year ago.

All PPPD patients were instructed for home based vestibular and balance exercises within in the scope of diagnosis in our outpatient clinic. In seven patients (27%) PPPD was treated using serotonergic medication (citalopram in *n* = 5; sertraline in *n* = 1; venlafaxine in *n* = 1). Most patients (*n* = 19, 73%) did not take any centrally active medication, the same accounted for all controls. A history of anxiety disorder was obtained in 4 PPPD patients (15%) of which two reported a history of coexisting depression (7.5%). Demographics and patient characteristics are summarized in Table 1.

**Table 1:** Demographics, clinical characteristics and scores in patients with persistent postural-perceptual dizziness and healthy controls

	PPPD Mean +/- SD M; [Min-Max]	HC Mean +/- SD M; [Min-Max]	Significance level ***p***
N	26	33	N/A
Gender (male/female)	0/26	0/33	n.a.
Age (years)	47.27 +/- 10.16 N/A; [29-64]	46.94 +/- 10.13 N/A; [28-65]	0.902
Disease duration (months)	64.73 +/- 65.93 N/A; [5-240]	N/A	
Caloric testing (deg/s)	88.5 +/- 31.7 91.5; [27.8-157.3]	N/A	
Normal physical and neuro-otologic examination N (%)	26 (100%)	33 (100%)	n.a.
Serotonergic medication N (%)	7 (26%)	0 (0%)	<0.001**
HADS-A	8.46 +/- 3.85 8; [1–17]	4.33 +/- 3.28 4; [0-15]	<0.001*
HADS-D	5.31 +/- 3.91 5; [1–13]	2 +/- 2.5 1; [0-10]	<0.001*
MSSQ raw	22.18 +/- 13.69 16.75; [4-48]	14.47 +/- 10.14 10; [4-33.88]	0.08*
STAI-S	46.5 +/- 10.98 44.5; 24-66]	32.18 +/- 7.23 32; [21-45]	<0.001*
STAI-T	44.31 +/- 11.07 43.5; [24-63]	32.91 +/- 7.8 31; [22-51]	<0.001*
SSAS	24.81 +/- 7.91 24; [11–40]	22.36 +/- 5.98 22; [12–39]	0.207*
WI	31.65 +/- 10.97 32.5; [14-53]	20.88 +/- 4.79 20; [14–35]	<0.001*
DHI	29.5 +/- 18.89 26; [10-64]	N/A	
VSS	33 +/- 16.54 33.5; [6-75]	N/A	

Significant group differences between persistent postural-perceptual dizziness (PPPD) patients and healthy controls (HC) are highlighted using bold type. If not annotated givenp values were calculated using Student’s t-test, additional statistical tests used were Mann-Whitney test* and Fisher’s Exact test**

*SD* Standard deviations, *N* numbers, *Min, Max* minimum and maximum values, *M* medians, Calculations not possible or not reasonable are marked as N/A. *DHI* Dizziness Handicap Inventory, *HADS* Hospital Anxiety and Depression Scale, *MSSQ* Motion Sickness Susceptibility Questionnaire, *SSAS* Somatosensory Amplification Scale, *STAI* State-Trait Anxiety Inventory, *VSS* Vertigo Symptoms Scale, *WI* Whitely Index

### Rotary chair test

**Fig. 1:**
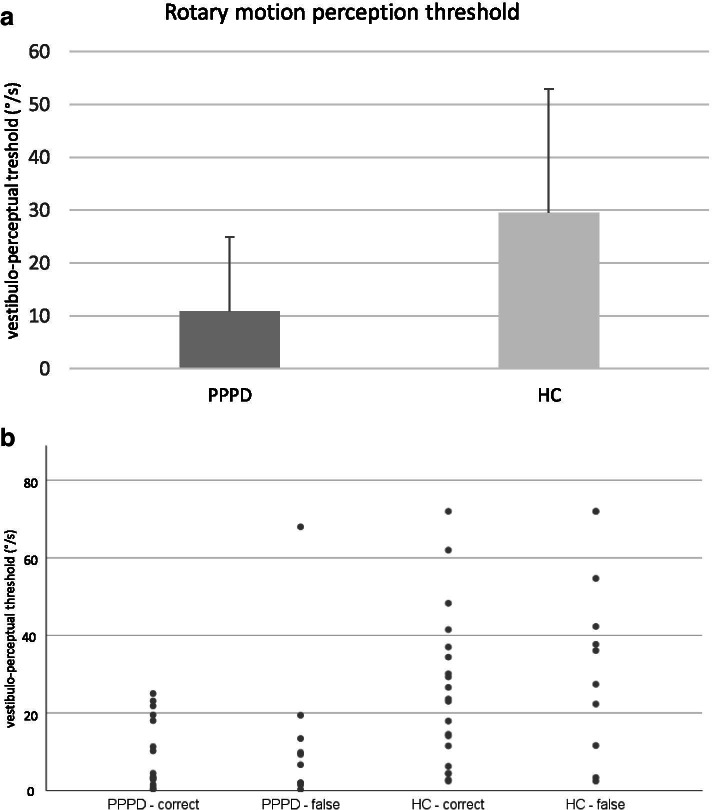
**a** Rotary motion perception threshold. Thresholds for initial rotary motion perception are shown for persistent postural-perceptual dizziness (PPPD) patients and healthy controls (HC). PPPD patients had a significantly lower threshold (PPPD: 10.85 +/-14.12 °/s; HC: 29.48 +/-23.49 °/s; *p*<0.001, Mann-Whitney test). Bars indicate standard error of mean. **b** Individual motion perceptual thresholds in PPPD patients and HC. Subjects’ data points for the individual motion perceptual thresholds are shown. In addition, the respective data of direction-specific perception of PPPD patients and HC are presented

Patients showed an increased sensitivity to the perception of initial rotary motion compared to healthy controls (10.9°/s vs. 29.5°/s; *p*<0.001, Mann-Whitney test), see Fig. 1a, b and Table 2. The extent of increased vestibular sensitivity was proportional to the duration of the disease (*r*=-0.4, *p*=0.043, Spearman rank) but did not relate to severity of dizziness handicap or psychological variables.

**Table 2:** Measurements of rotary chair experiment

	PPPD Mean +/- SD M; [Min-Max]	HC Mean +/- SD M; [Min-Max]	Significance level ***p***
Rotary motion perception threshold (°/s)	10.85 +/- 14.12 N/A; [0.3-68.0]	29.48 +/- 23.49 N/A; [2.5-72.0]	<0.001*
Onset (in seconds) of sickness rating phases during rotation …			
Initial vegetative symptoms (SR 2) (s)	283.19 +/- 145.06 N/A; [62-573]	683.9 +/- 250.78 N/A; [277-1166]	<0.001*
Mild nausea (SR 3) (s)	503.46 +/- 175.56 N/A; [215-740]	704.5 +/- 126.57 N/A; [615-794]	0.233*
Severe vegetative symptoms (SR 4) (s)	719.95 +/- 238.39 N/A; [247-1195]	739 +/- 0 N/A; [739-739]	0.939*
Duration of sickness rating phases during rotation …			
Asymptomatic (SR 1) (s)	283.19 +/- 145.06 N/A; [62-573]	1043.61 +/- 275.14 N/A; [277-1200]	<0.001*
Initial vegetative symptoms (SR 2) (s)	220.27 +/- 139.49 N/A; [52-637]	417 +/- 277.89 N/A; [34-923]	0.008*
Mild nausea (SR 3) (s)	308.81 +/- 187.76 N/A; [32-588]	265 +/- 199.4 N/A; [124-406]	0.809*
Recovery from vegetative symptoms (SR) … after completion of rotation			
… 1 min	2.65 +/- 0.8 2; [1–4]	1.18 +/- 0.39 1; [1, 2]	<0.001*
… 5 min	1.65 +/- 0.8 1; [1–4]	1 +/- 0 1; [1]	<0.001*
…10 min	1.12 +/- 0.43 1; [1–3]	1 +/- 0 1; [1]	0.11*
… 15 min	1.04 +/- 0.2 1; [1, 2]	1 +/- 0 1; [1]	N/A
… 20 min	1 +/- 0 1; [1]	1 +/- 0 1; [1]	N/A
Beginning of per-rotatory nystagmus (s)	9.11 +/- 9.6 [1-36]	5.2 +/- 6.65 [1-26]	0.159
Maximum velocity of the slow phase (deg/s)	39.27 +/- 26.97 [3.5-92.1]	45.96 +/- 40.17 [6.0-149.6]	0.85
Average velocity of slow phase (deg/s)	8.26 +/- 5.46 [1.9-25.7]	8.95 +/- 7.17 [3.0-29.3]	0.92

Significant group differences between PPPD patients and HC are highlighted using bold type. If not annotated given *p* values were calculated using Student’s t-test, additional statistic test used: Mann-Whitney test*. As there were no interocular differences regarding the nystagmus measurements for the sake of clarity these exemplary are only given for the left eye

*SD* Standard deviations, *N* numbers, *Min, Max* minimum and maximum values, *M* medians; Calculations not possible or not reasonable are marked as N/A. *Deg* degree, *min* minute, *Nyst* nystagmus, *SR* sickness rating, *s* seconds

The error rate of direction specific perception of chair rotation was not significantly different between PPPD and HC (38.5% vs. 33.3%; *p*=0.905, Fisher’s exact). To additionally check if patients with very low latencies differ from those with higher thresholds in this regard, we compared the 25% of patients with the shortest latency (≤ 3.3°/s) with the rest of patients. We did not observe any statistical difference regarding chance of correct decision (66,6% vs. 60%, *p* = 1.000, Fisher exact test).

PPPD patients also showed marked differences in development of motion sickness during and after rotation. In the patient group, no one remained symptom-free over the 20-minute duration of the examination, whereas 23 of 33 controls (69.7%) remained symptom-free. 10 controls (30.3%) entered SR phase 2, two controls (6.1%) entered SR phase 3 and only one person (3.0%) suffered severe symptoms (phase 4). The entry into SR phase 4 triggered the interruption of the rotary motion before the end of the 20 minutes to keep distress for the participants to a minimum. In contrast, all PPPD patients entered SR phase 2 and SR phase 3 (100%) and 21 patients even reached SR phase 4 (80.8%). Whereas the interruption of the rotational movement (=SR 4) occurred in only one healthy control.

**Fig. 2:**
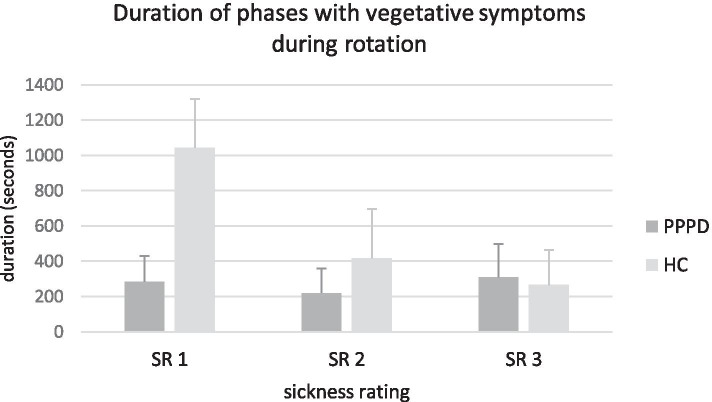
Duration of phases with vegetative symptoms during rotation. Mean duration of sickness rating phases (SR 1-3) are shown during rotation. PPPD patients develop vegetative symptoms earlier as they pass through the different phases with vegetative symptoms faster (SR 1; PPPD: 283.19 s +/-145.06; HC: 1043.61 s +/-275.14; *p*<0.001, Mann-Whitney test; SR 2; PPPD: 220.27 s +/-139.49; HC: 417 s +/-277.89; *p*=0.008, Mann-Whitney test). Duration of SR 3 did not differ significantly (PPPD: 308.81 s +/-187.76; HC: 265 +/-199.4; *p*=0.809, Mann-Whitney test). SR 4 is not illustrated as the entry in this phase leads to the interruption of the rotation. Bars indicate standard error of mean

Similar accounts for the duration of symptoms. The asymptomatic phase (duration of SR 1; *p*<0.001, Mann-Whitney test) and the phase with initial vegetative symptoms (duration of SR 2; *p*=0.008, Mann-Whitney test) was significantly shorter in patients compared to control subjects, see Fig. 2.

**Fig. 3:**
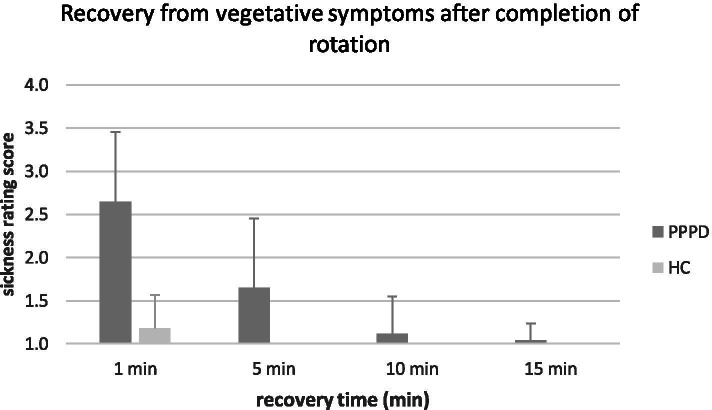
Recovery from vegetative symptoms after completion of rotation. After completion of rotation PPPD patients recovered significantly slower than healthy controls (HC) from vegetative symptoms (sickness rating score = SR) (mean SR: minute 1; PPPD: 2.65 +/-0.8, HC: 1.18 +/-0.39; *p*<0.001 and minute 5; PPPD: 1.65 +/-0.8, HC: 1 +/-0; *p*<0.001, Mann-Whitney test). Since all HC were symptom-free from minute 5 after completion (SR 1), no bars and error bars appear for HC from minute 5 to 15. After 20 minutes all participants were again asymptomatic, therefore this is not illustrated

Further differences were seen for the recovery from vegetative symptoms. After completion of rotation, patients showed significantly more vegetative symptoms in comparison to healthy controls after both minute 1 (*p*<0.001, Mann-Whitney test) and minute 5 (*p*<0.001, Mann-Whitney test), see Table 2. After 5 minutes all controls were symptom-free, whereas 50% of patients still suffered from nausea or dizziness, see Fig. 3. After a recovery period of 20 minutes, all participants were again symptom-free.

### Vestibulo-ocular threshold

The vestibulo-ocular threshold did not differ significantly between the PPPD patients and healthy controls (9.11 s ±9.6 vs 5.2 s ±6.65; *p*=0.159, t-test). Furthermore, the average (9.2°/s vs 7.9°/s; *p*=0.48, t-test) and maximum (45.4°/s vs 38.3°/s; *p*=0.44, t-test) velocity of the slow phase of the per-rotatory nystagmus did not differ between healthy controls and PPPD patients, see Table 2.

### Functional and psychometric variables

Severity of vertigo and dizziness and its influence on daily life of patients was measured by scores of VSS and DHI (see Table 1). In PPPD patients the DHI scores were 29.5 ±18.89 and the VSS scores were 33.0 ±16.54 indicating mild to moderate handicaps.

Regarding depressive symptoms higher values were observed in PPPD patients compared to controls in HADS-D-score (5.31 ±3.91 vs. 2.0 ±2.5; *p*<0.001, Mann-Whitney test) and in anxiety measures HADS-A (8.46 ±3.85 vs. 4.33 ±3.28; *p*<0.001, Mann-Whitney test) as well as in STAI-S (46.5 ±10.98 vs. 32.18 ±7.23; *p*<0.001, Mann-Whitney test) and STAI-T (44.31 ±11.07 vs. 32.91 ±7.8; *p*<0.001, Mann-Whitney test).

To investigate the potential role of depression on group differences, we performed correlation analyses using the HADS-score, but there was no significant effect on vestibular perception or vegetative symptoms. For detail of correlation analysis see Supplementary Table S1.

Using the anxiety scores (STAI) for correlation analyses there was an effect on vegetative symptoms but not on vestibular perception threshold. Patients with PPPD showed vegetative symptoms (SR 3) earlier as mean subjective anxiety scales increased (STAI-S: *r*=-0.4, *p*=0.043, Spearman rank; STAI-T: *r*=-0.426, *p*=0.03, Spearman rank).

Furthermore, according to the Whitely Index, patients with PPPD were overall more concerned about their health (31.65 ±10.97 vs. 20.88 ±4.79; *p*<0.001, Mann-Whitney test), whereas there was no difference in the SSAS (24.81 ±7.91 vs. 22.36 ±5.98; *p*=0.207, Mann-Whitney test) compared to healthy controls. Correlation analysis showed an inverse correlation of the severity of somatosensory amplification with the beginning of mild and moderate vegetative symptoms during rotation (SR 2, *p*=0.002, Spearman rank; SR 3, *p*=0.039, Spearman rank).

The mean MSSQ raw score did not differ significantly between PPPD patients and controls (22.18 ±13.69 vs. 14.47 ±10.14; *p*=0.08, Mann-Whitney test).

Correlation analysis in healthy controls were performed for SSAS, HADS, Whitely Index, STAI and MSSQ and remained without significant findings.

## Discussion

Patients with PPPD showed a reduced vestibular perception threshold and expressed more vegetative symptoms during and after rotation in the rotary chair experiment compared to healthy controls. These objective perception threshold deficits and increased vegetative responses to a vestibular stimulus in a functional vestibular disorder add to the current understanding of PPPD. With regard to clinical characteristics, disease duration, mean scores of DHI, HADS and STAI and the percentage of patients with a peripheral vestibular disorder as the suspected triggering event for PPPD are consistent with the current consensus criteria of the Bárány Society and published reports of patients with PPPD [5, 6].

Perceptual thresholds consecutively improve, as the sinusoidal rotation frequency increases over 0.2 Hz, and plateau at about 0.5 Hz [22]. This suggests that vestibular signals may undergo a high-pass filtering. This physiological threshold may be altered by psychological influences (i.e. anxiety, body vigilance) as suggested for visual cues during sensory integration previously [23]. Based on increased comorbidity Balaban et al. suggested a concept for balance and anxiety in the context of migraine [24]. He hypothesized that vestibular processing and anxiety share central pathways to the amygdala and cerebral cortex [25]. Similar may apply for PPPD as anxiety here also is frequently comorbid [5], and increased connectivity within brain networks regulating interoception, emotional behavior and cognitive control in patients with functional vestibular disorder was previously demonstrated [26, 27]. In the current study patients showed a correlation between anxiety measure (STAI) and vegetative symptoms during the chair rotation. This sensitized vegetative response pattern may reflect one aspect of the affective mediation of vestibular processing in PPPD. However, PPPD and control groups had mean HADS-D-scores in the normal range and the mean HADS-A-score for the PPPD group was barely in the clinically meaningful range, similar accounts for the STAI.

Motion perception can be altered by psychological factors. In a different experiment subjects were asked to simply imagine themselves rotating in a chair before the actual rotation began. Vestibular perceptual thresholds were lower when the real rotations and the imagined rotations had the same direction, as were thresholds for the vestibular ocular reflex [28]. This finding indicates that top-down modulation of low-level vestibular perception to physical rotation is possible by psychological influence alone. Thus, the vestibular system appears to be susceptible to modulation by higher order cognitive processes associated with attention, working memory or mental imagery, and potentially anxiety and expectation [29, 30].

A neuroscientific mechanism is currently lacking as to why some individuals rely more heavily upon visual cues [23], and others on vestibular (i.e. gravito-inertial cues) or somato-proprioceptive cues [31]. The development of PPPD in certain patients may be related to a psychological trait in patients with predominant vestibular perception preference. Structural and functional imaging studies suggested an abnormal integration of visual and vestibular information as well as alterations in multisensory vestibular brain areas [26, 32–35]. This supports the assumption that alterations of vestibular processing in PPPD are based on widespread changes of the multisensory vestibular system. The affection of somatosensory perception was demonstrated by the detection of a habituation deficit in patients with PPPD following trigeminal pain stimuli at brainstem level applied with the nociceptive blink reflex [19]. Galvanic vestibular stimulation showed a reduced perception threshold for body motion in PPPD patients, which was attributed to a lowered sensory feedback control [36]. Our data imply reduced motion perception thresholds, which may be attributed to cortical and subcortical overexcitability and may explain the abnormal self-motion awareness. This may also lead to exacerbations of the subjective postural instability, which improve under cognitive distraction [37]. Our findings may also support the concept of increased attentional effects on vestibular processing in PPPD. This is in line with the current consensus criteria, which explicitly allows subtyping of this multifaceted clinical entity (e.g. posturally predominant subtype, visually predominant subtype) [6]. Therefore, it appears most likely that patients with PPPD have a multimodal dysfunction not limited to vestibular stimuli alone.

A change of the vestibular sensitivity is not specific to PPPD, but can also be found in other vertigo associated disorders, such as vestibular migraine [20] and motion sickness [38]. This increased sensitivity of the vestibular system was hypothesized as correlate for a habituation deficit or cortical hyperexcitability. Kinetosis could be interpreted as a sensitivity threshold for the perception of vestibular stimuli that is adjusted too finely.

Our study has some limitations. The self-report instrument HADS measured a slightly higher rate of anxiety values in PPPD group. Nevertheless, we did not find evidence for fulfilling the diagnostic criteria for active anxiety disorder in these patients. This is in concordance with previous research, patients with functional dizziness score elevated anxiety values even though they do not suffer from anxiety disorder [39, 40]. However, the increased level of psychological factors in the PPPD group and the proportion of patients taking serotonergic medication as PPPD treatment could have influence to the perception threshold. However, the direction of this alteration could be contrary, as an anxious mode of vestibular control may decrease the perception threshold, while selective serotonin-reuptake inhibitors most likely increase it and are therefore used in this indication [5, 25, 41]. Our relatively small number of patients is a limitation, but we decided to exclude PPPD patients with clinically significant comorbid anxiety disorder or depression to prevent bias. In contrast to PPPD in general, we only included female participants, this should not influence the results, as there were no differences of vestibular perceptual thresholds between males and females reported in a previous investigation [42]. Ultimately, an influence on these study results cannot be excluded here.

One could argue that exclusion criteria of the control group were too strict and thereby this group is not reflecting the general population , as vertigo and depression were ruled out although prevalent in general population. However, in order to shed light on the pathophysiology, a comparison to a healthy group seems appropriate, especially since an otherwise necessary exact matching in terms of comorbid disorders appears not obtainable.

 It remains an open discussion which stimulation technique is the best for such a chair rotation study. Some authors prefer a repeated stimulation to assess discrimination thresholds [43]. However, investigating participants once has the advantage of avoiding erroneous measurements due to within-session response dynamics induced by repetitive stimuli. Moreover, the protocol used here is also suitable to evaluate the perceptual detection threshold and additionally the development of vegetative symptoms accurately [21]. Furthermore, this simplified and well-tolerated protocol may be used to measure therapy effects in the sense of a longitudinal study.

Concerning the debate as to whether PPPD should be termed psychogenic or functional, we think that both terms can be used depending on the clinical and individual situation of the patient. However, we find the term functional most suitable in cases when there is no identifiable psychological cause, the patient is accepting the diagnosis, and symptoms are interfering with everyday activities.

## Conclusions

Taken together, our study shows that patients with PPPD have alterations in vestibular processing which can be objectified. They appear to be more sensitive to vestibular stimuli with pronounced vegetative reaction and lower detection thresholds parallel to an increasing illness duration. In PPPD these abnormalities are likely to reflect chronic maladaptation and dysmodulation of the vestibular system in terms of an increased vestibular excitability or deficient habituation leading to motion intolerance and induction of dizziness when exposed to movement. The exact underlying cause for this phenomenon remains unclear and further systematic research is required.

## Supplementary Information

**Additional file 1: Table S1**. Correlation analyses of vestibular parameters and psychometric variables in PPPD.

## Data Availability

The datasets analysed during the current study are available from the corresponding author on reasonable request.

## Abbreviations

CSD: Chronic subjective dizziness
DHI: Dizziness Handicap Inventory
HADS: Hospital Anxiety and Depression Scale
HC: Healthy controls
MSSQ: Motion Sickness Susceptibility Questionnaire
PPPD: Persistent postural-perceptual dizziness
PPV: Phobic postural vertigo
SR: sickness rating
SSAS: Somatosensory Amplification Scale
STAI: State-Trait Anxiety Inventory
VM: Vestibular migraine
VOR: Vestibulo-ocular reflex
VSS: Vertigo Symptoms Scale
WI: Whitely Index
