# Supplementary material for: Reduced vestibular perception thresholds in persistent postural-perceptual dizziness- a cross-sectional study
Source: BMC Neurol. 2021 Oct 12;21:394. doi: 10.1186/s12883-021-02417-z (PMC8507224; doi:10.1186/s12883-021-02417-z)
Supplement: Supplementary file 1 — Additional file 1: Table S1. Correlation analyses of vestibular parameters and psychometric variables in PPPD. [file 12883_2021_2417_MOESM1_ESM.docx]

**Reduced vestibular perception thresholds in persistent postural-perceptual dizziness- a cross-sectional study**

**Sebastian Wurthmann^1^, Dagny Holle^1^, Mark Obermann^3^, Miriam Roesner^1^, Michael Nsaka^1^, Armin Scheffler^1^, Christoph Kleinschnitz^1^, Steffen Naegel^1,2^**

**^1^**Department of Neurology and Dizziness and Vertigo Center Essen, University of Duisburg-Essen, Germany

**^2^**Department of Neurology, Martin-Luther-University Halle-Wittenberg, Halle/Saale, Germany

**^3^**Department of Neurology, Weser-Egge Hospital Höxter, and University of Duisburg-Essen, Germany

**Table S1: Correlation analyses of vestibular parameters and psychometric variables in PPPD.**

|  | | **DHI** | **VSS** | **SSAS** | **WI** | **STAI-S** | **STAI-T** | **MSSQ** | **HADS-A** | **HADS-D** | **DD** |
| --- | --- | --- | --- | --- | --- | --- | --- | --- | --- | --- | --- |
| **Vestibular-perceptual threshold (°/s)** | r | -0.078 | -0.246 | -0.316 | -0.084 | -0.074 | -0.240 | -0.192 | -0.205 | -0.112 | -0.399 |
|  | p | 0.706 | 0.225 | 0.116 | 0.682 | 0.719 | 0.237 | 0.477 | 0.314 | 0.585 | **0.043** |
| **Time elapsed until respective sickness rating was reached ...** | | | | | | | | | | | |
| **… SR 2 during rotation** | r | -0.107 | -0.198 | -0.585 | -0.207 | -0.186 | -0.349 | -0.041 | -0.302 | -0.095 | -0.017 |
|  | p | 0.604 | 0.333 | **0.002** | 0.310 | 0.364 | 0.080 | 0.879 | 0.134 | 0.643 | 0.933 |
| **… SR 3 during rotation** | r | -0.151 | -0.118 | -0.406 | -0.244 | -0.400 | -0.426 | 0.080 | -0.366 | -0.224 | 0.050 |
|  | p | 0.461 | 0.566 | **0.039** | 0.229 | **0.043** | **0.030** | 0.769 | 0.066 | 0.272 | 0.808 |
| **… SR 4 during rotation** | r | 0.113 | 0.027 | 0.087 | -0.223 | -0.289 | -0.209 | 0.421 | -0.138 | 0.050 | 0.022 |
|  | p | 0.625 | 0.909 | 0.709 | 0.331 | 0.203 | 0.362 | 0.152 | 0.550 | 0.828 | 0.926 |
| **Duration of sickness rating phases during rotation …** | | | | | | | | | | | |
| **… asymptomatic  (SR 1)** | r | -0.107 | -0.198 | -0.585 | -0.207 | -0.186 | -0.349 | -0.041 | -0.302 | -0.095 | -0.017 |
|  | p | 0.604 | 0.333 | **0.002** | 0.310 | 0.364 | 0.080 | 0.879 | 0.134 | 0.643 | 0.933 |
| **… initial symptoms  (SR 2)** | r | -0.004 | 0.080 | 0.077 | -0.286 | -0.366 | -0.206 | 0.260 | -0.161 | -0.182 | 0.051 |
|  | p | 0.985 | 0.699 | 0.708 | 0.157 | 0.066 | 0.312 | 0.332 | 0.431 | 0.374 | 0.804 |
| **… mild nausea  (SR 3)** | r | 0.104 | 0.002 | 0.259 | -0.021 | -0.141 | -0.149 | 0.339 | -0.026 | -0.150 | -0.196 |
|  | p | 0.612 | 0.994 | 0.201 | 0.921 | 0.493 | 0.467 | 0.198 | 0.899 | 0.465 | 0.337 |
| **Recovery from vegetative symptoms (SR) … after completion of rotation** | | | | | | | | | | | |
| **… 1 min** | r | 0.184 | 0.148 | 0.355 | 0.030 | 0.121 | 0.221 | -0.099 | 0.173 | 0.033 | -0.218 |
|  | p | 0.368 | 0.471 | 0.075 | 0.884 | 0.555 | 0.279 | 0.714 | 0.397 | 0.874 | 0.285 |
| **… 5 min** | r | 0.023 | -0.026 | 0.249 | -0.016 | 0.080 | 0.174 | -0.257 | 0.166 | -0.065 | -0.279 |
|  | p | 0.912 | 0.901 | 0.220 | 0.940 | 0.697 | 0.394 | 0.337 | 0.419 | 0.752 | 0.168 |
| **… 10 min** | r | -0.036 | -0.113 | -0.117 | 0.284 | 0.358 | 0.324 | -0.500 | 0.065 | 0.169 | 0.223 |
|  | p | 0.863 | 0.583 | 0.568 | 0.160 | 0.073 | 0.106 | 0.049 | 0.751 | 0.408 | 0.273 |
| **… 15 min** | r | 0.281 | 0.053 | 0.134 | 0.294 | 0.227 | 0.280 | -0.253 | 0.268 | 0.230 | 0.307 |
|  | p | 0.165 | 0.796 | 0.515 | 0.145 | 0.264 | 0.165 | 0.345 | 0.185 | 0.259 | 0.127 |
| **DD** | r | -0.012 | -0.078 | 0.061 | 0.271 | 0.215 | 0.356 | -0.330 | 0.391 | 0.332 | 1.000 |
|  | p | 0.954 | 0.704 | 0.767 | 0.180 | 0.291 | 0.074 | 0.212 | **0.048** | 0.097 | N/A |

**Table S1.** Significant correlations in PPPD patients are highlighted using bold type. Correlation analyses were carried out using Spearman rank correlation, as these data were not normally distributed. Calculations not possible or not reasonable are marked as N/A.

DD=disease duration; DHI=Dizziness Handicap Inventory; HADS=Hospital Anxiety and Depression Scale; MSSQ= Motion Sickness Susceptibility Questionnaire; SR=sickness rating; SSAS=Somatosensory Amplification Scale; STAI=State-Trait Anxiety Inventory; VSS=Vertigo Symptoms Scale; WI=Whitely Index.
